# 2-NBDG Uptake in *Gossypium hirsutum in vitro* ovules: exploring tissue-specific accumulation and its impact on hexokinase-mediated glycolysis regulation

**DOI:** 10.3389/fpls.2023.1242150

**Published:** 2023-09-25

**Authors:** Melina Shamshoum, Ofir Aharon Kuperman, Sapir Korman Shadmi, Maxim Itkin, Sergey Malitsky, Filipe Natalio

**Affiliations:** ^1^ Department of Plant and Environmental Sciences, Weizmann Institute of Science, Rehovot, Israel; ^2^ Metabolic Profiling Unit, Life Sciences Core Facilities, Weizmann Institute of Science, Rehovot, Israel

**Keywords:** cotton, ovules, hexokinases, 2-NBDG, metabolism, glycolysis

## Abstract

Fluorescent glucose derivatives are valuable tools as glucose analogs in plant research to explore metabolic pathways, study enzyme activity, and investigate cellular processes related to glucose metabolism and sugar transport. They allow visualization and tracking of glucose uptake, its utilization, and distribution within plant cells and tissues. This study investigates the phenotypic and metabolic impact of the exogenously fed glucose derivative, 2-(N-(7-nitrobenz-2-oxa-1,3-diazol-4-yl)amino)-2-deoxyglucose) (2-NBDG) on the fibers of *Gossypium hirsutum* (Upland cotton) ovule *in vitro* cultures. The presence of 2-NBDG in the culture medium did not lead to macroscopic morphological alterations in ovule and fiber development or to the acquisition of fluorescence or yellow coloration. Confocal laser scanning microscope imaging and chromatographic analysis of cotton ovules’ outer rim cross-sections showed that the 2-NBDG is transported from the extracellular space and accumulated inside some outer integument cells, epidermal cells, and fertilized epidermal cells (fibers), but is not incorporated into the cell walls. Untargeted metabolic profiling of the fibers revealed significant changes in the relative levels of metabolites involved in glycolysis and upregulation of alternative energy-related pathways. To provide biochemical and structural evidence for the observed downregulation of glycolysis pathways in the fibers containing 2-NBDG, kinetics analysis and docking simulations were performed on hexokinase from *G. hirsutum* (GhHxk). Notably, the catalytic activity of heterologously expressed recombinant active GhHxk exhibited a five-fold decrease in reaction rates compared to D-glucose. Furthermore, GhHxk exhibited a linear kinetic behavior in the presence of 2-NBDG instead of the Michaelis-Menten kinetics found for D-glucose. Docking simulations suggested that 2-NBDG interacts with a distinct binding site of GhHxk9, possibly inducing a conformational change. These results highlight the importance of considering fluorescent glucose derivatives as ready-to-use analogs for tracking glucose-related biological processes. However, a direct comparison between their mode of action and its extrapolation into biochemical considerations should go beyond microscopic inspection and include complementary analytical techniques.

## Introduction

Glucose metabolism is a fundamental process for maintaining cellular homeostasis and providing energy for the growth and development of organisms. In plants, glucose serves as a central building block for the synthesis of complex carbohydrates, including cellulose, hemicellulose, and pectin, which collectively form the cell wall (York et al., 1986; Heredia et al., 1995). Understanding glucose metabolism is of paramount importance for advancing our knowledge of plant physiology, sugar transport (source-sink relations), and potentially, developing strategies to improve crop productivity and quality (Vorwerk et al., 2004; Santiago et al., 2013; Geoghegan et al., 2017).

In recent years, the use of exogenous fluorescent glucose derivatives has emerged as a powerful tool for investigating cellular processes in plants (Wang and Ruan, 2012), such as elucidating sugar transport mechanisms (Knoblauch et al., 2015; Rottmann et al., 2018; Rottmann and Stadler, 2019). However, glucose derivatives are considered to be analogs of glucose that possess similar structural properties but often exhibit altered chemical characteristics and interactions with the organisms (Jang and Sheen, 1994; Kurtoglu et al., 2007; Fatangare et al., 2015; Fatangare and Svatoš, 2016; Van Scherpenzeel et al., 2022). Among these derivatives, 2-(N-(7-nitrobenz-2-oxa-1,3-diazol-4-yl)amino)-2-deoxyglucose (2-NBDG) was first used to study the glucose analogs uptake and intracellular metabolization by *Escherichia coli* (Yoshioka et al., 1996a; Yoshioka et al., 1996b). In *E. coli*, 2-NBDG is imported by glucose transporters into the cytosol with K_m_ values similar to those reported for carbon-isotope-labeled glucose tracers (Yoshioka et al., 1996b). There, 2-NBDG is phosphorylated by the glycolytic enzyme hexokinase in position C6 to produce the phosphorylated derivative 2-NBDG 6-phosphate (Yoshioka et al., 1996a).

The utilization of 2-NBDG in plants provided valuable insights into the mechanisms involved in glucose uptake into heterotrophic plant cells. In the case of *Acer pseudoplatanus L.* (sycamore) cell cultures, two potential pathways were identified for the intracellular transportation of 2-NBDG: a saturable carrier-mediated mechanism and a non-saturable, presumably endocytic transport process as proposed by Etxeberria and colleagues (Etxeberria et al., 2005). In another study conducted on *Olea europaea* (common olive), it was suggested that non-saturable 2-NBDG uptake is mediated by a protein transporter. This transporter is hindered by mercury chloride, and exhibits a lower affinity to 2-NBDG compared to natural D-glucose (Conde et al., 2007). Additionally, in *Solanum lycopersicum* (tomato) lines that overexpress MdHT1.2 (a hexose transporter from apple *Malus × domestica Borkh*), 2-NBDG was employed to observe the uptake of sugars by the roots (Tian et al., 2023).

Cotton (Genus *Gossypium*) is a globally significant crop renowned for its valuable fibers, which are extensively utilized in the textile industry. Unraveling the intricate mechanisms that govern cotton fiber development is of utmost importance (Delmer et al., 1977; Carpita and Delmer, 1981; Haigler et al., 1991; Haigler et al., 2001; Haigler et al., 2005; Haigler et al., 2012; Sethaphong et al., 2013; Haigler and Roberts, 2019). In this context, the fertilized cotton ovule *in vitro* cultures has emerged as a valuable model system for studying the biochemistry and physiology involved in the formation of cotton fibers (Beasley, 1973; Beasley and Ting, 1974; Triplett, 2000; Natalio et al., 2017).

## Materials and methods

### Cotton growth conditions and fertilized ovule *in vitro* cultures

Cotton (*Gossypium hirsutum L.* Upland cotton) was grown in soil in a greenhouse under controlled temperature and humidity conditions (25 ± 5°C, 40% RH) (Natalio et al., 2016). The fiber quality was described elsewhere (Stiff and Haigler, 2012; Ijaz et al., 2019). Flowers were harvested two days post-anthesis (2 dpa). Ovaries were exposed using a surgical steel scalpel and sterilized in a solution of NaOCl (6%) for 2 minutes at room temperature. Then, the fertilized ovules were aseptically removed and placed floating onto sterile Beasley and Ting (BT) medium (50 mL) supplemented with gibberellic acid (GA, 5 µM, Merck, Germany) and indoleacetic acid (IAA, 0.5 µM, Merck, Germany) (Beasley, 1973; Beasley and Ting, 1974). The cultures were kept in the dark at 30°C and 5% CO_2_ for 20 days.

### Fertilized cotton ovule *in vitro* cultures and exogenous feeding of a glucose derivative

2-(N-(7-nitrobenz-2-oxa-1,3-diazol-4-yl)amino)-2-deoxyglucose) (2-NBDG, ThermoFisher Scientific, Israel) (1 µM) was resuspended in sterile BT medium containing phytohormones (5 µM of GA and 0.5 µM IAA) (Beasley, 1973; Beasley and Ting, 1974). The cultures were kept in the dark at 30°C and 5% CO_2_ for 20 days. Following the growth period, the fibers were carefully removed with a sterile scalpel, washed eight times with double distilled water (DDW), and placed at - 80°C until further characterization. All experiments were carried out in triplicate.

### Cross-section preparation and confocal laser scanning microscopy imaging

The cotton ovules were carefully removed, transferred to a plate containing double-distilled water to wash excess medium, and then placed on filter paper to soak. Then, a razor blade was used to perform freehand cross-sections of the ovules. Images from the freshly prepared cross-section were acquired using a Nikon A1R HD25 confocal laser scanning microscope, mounting an A1-DUVB-2 GaAsP detector, a 489 nm laser, and a 60X water objective. All images were collected in the 520-530 nm range for both FITC emission and transmitted light, using a power of 5 mW, a gain of 80 seconds, and submicrometric spatial resolution. The NIS-Element Ar software (NIS Elements, imaging software, v 5.01, Japan) was employed for data collection. Data was processed using Fiji image processing software v 1.53t (Schindelin et al., 2012).

### Metabolite extraction from fibers excised from cotton ovule *in vitro* cultures

Polar metabolite extraction was performed as previously described elsewhere (Malitsky et al., 2016) with some modifications. Lyophilized tissues were ground with metal beads for 2 min at 25 Hz using a MM 400 Mixer mill (Retsch, Germany), and the resulting powder was extracted with 1 mL of a pre-cooled (- 20°C) homogenous methanol: methyl-tertbutyl-ether (TMBE) 1:3 (v/v) mixture. The tubes were vortexed and then sonicated for 30 min in a sonication bath (Branson B321 ultrasonic cleaner), taken for a brief vortex every 10 min. to avoid overheating of the samples. Ice was added to the sonication bath. Then, DDW: methanol (3:1) (v/v) solution (0.5 mL) was added to the tubes, followed by centrifugation (15300 rcf, 10 min, room temperature). The upper organic phase was discarded. The polar phase was re-extracted as described above, with 0.5 mL of TMBE. The polar phase used for polar metabolite analysis was stored at - 80°C until further analysis. For analysis, the samples were resuspended in 150 µL of DDW:methanol (1:1), centrifuged to remove debris (15,300 rcf, 10 min, room temperature), and the supernatant was transferred into HPLC vials.

### Liquid chromatography-mass spectrometry analysis of polar metabolites extracted from fibers excised from cotton ovule *in vitro* cultures

Metabolic profiling of the polar phase was done as described elsewhere with minor modifications. (Zheng et al., 2015) Briefly, analysis was performed using an Acquity I class UPLC System combined with a mass spectrometer (Thermo Exactive Plus Orbitrap, Israel) operated in a negative ionization mode. The LC separation was done using SeQuant Zic-pHilic (150 mm × 2.1 mm, Merck, Israel) with a SeQuant guard column (20 mm × 2.1 mm, Merck, Israel). The mobile phase B: acetonitrile and mobile phase A: 20 mM ammonium carbonate with 0.1% ammonia hydroxide in water. The flow rate was kept at 200 μL min^-1^, and the gradient was as follows: 0 - 2 min 75% of B, 17 min 12.5% of B, 17.1 min 25% of B, 19 min 25% of B, 19.1 min 75% of B, and 19 min 75% of B.

### Identification of polar metabolites and data analysis

Data processing was done using TraceFinder software (Thermo Fisher, Israel). Detected compounds were identified by retention time and mass fragments against an in-house mass spectra library. Results were normalized using sample weight.

### Pathway analysis

Pathway analysis of the statistically significant metabolites was carried out in the Kyoto Encyclopedia of Genes and Genomes (KEGG)(Kanehisa et al., 2016). The organism selected was *Gossypium hirsutum* (ghi). Heatmaps were generated using OriginLabPro 2022 v9.9.0.225.

### Genome screening for hexokinases from *Gossypium hirsutum*

Screening of enzyme sequences from *Gossypium hirsutum* was carried out using the Kyoto Encyclopedia of Genes and Genomes (KEGG, organism KEEG code: ghi) directly linked to the fully sequenced genome (Li et al., 2015; Kanehisa et al., 2016; Hu et al., 2019). *G. hirsutum* is a tetraploid organism (4 genomes, AADD; 2n = 52) (Li et al., 2015). The protein and gene sequences of hexokinases (GhHxk) were retrieved from the KEGG database(Kanehisa et al., 2016). The amino acid sequences were aligned using Clustal Omega (https://www.ebi.ac.uk/Tools/msa/clustalo/with default settings). The generated phylogenetic trees were visualized using Interactive Tree of Life version 6.5.8 (https://itol.embl.de/).

### Heterologous expression in *Escherichia coli* and purification of recombinant *Gossypium hirsutum* hexokinase

The *G. hirsutum* gene for hexokinase was selected based on the phylogenetic proximity with wild-type *Arabidopsis thaliana* (Col-0) hexokinase 1 (AtHxk) (Feng et al., 2015), codon-optimized, produced synthetically by TwistBiosciences (USA), and cloned into pET-28-14His-bdSumo vectors containing a kanamycin-resistance cassette (Davidi et al., 2020). Our particular construct appends a His-tagged SUMO domain to the N-terminus of the protein, which promotes protein stability in *Escherichia coli* and standard Ni-NTA His-affinity purification. Furthermore, a suitable peptidase was used to cleave the protein from the His tag at the Sumo recognition site to yield high-purity tagless wild-type proteins (Peleg and Unger, 2008). Then, *E. coli* BL21(DE3) heat-shock competent cells were transformed using synthetic genes-pET-28-14His-bdSumo vector, incubated at + 37°C, 250 rpm in 5 mL of Luria-Broth (LB) medium supplemented with 50 µg/mL kanamycin overnight. Next, cultures were diluted (1:100) into 20 mL LB with 50 µg/mL kanamycin. The protein expression was induced when cells reached an OD_600_ of 0.8 by adding 0.2 mM isopropyl β-D-thiogalactoside (IPTG, Sigma, Israel) and incubated at + 16°C for 16 h. For protein extraction and purification, cells were harvested by centrifugation (4000 x g at + 4°C for 15 min), pellets were resuspended in 20 mM Tris buffer pH 7.5, 50 mM NaCl, 5 mM imidazole, and then lysed using a sonicator. Crude extracts were centrifuged for 30 min at 10,000 x g at + 4°C to remove the insoluble fraction. The soluble fraction was transferred to 1.5 mL tubes. A nickel magnetic bead system (PureProteomeTM; Millipore) was used for washing and binding following the manufacturer’s protocol. The recombinant enzymes were eluted by on-bead cleavage of the SUMO tag with bdSENP1 protease [400 µL of cleavage buffer, 20 mM Tris pH 7.5 + 50 mM NaCl containing bdSENP1 protease (8 µg/mL)] for 30 min at + 24°C under gentle agitation (250 rpm) (Frey and Görlich, 2014). Purified protein was separated from the tag-bounded magnetic beads using a magnetic rack and stored at + 4°C. Protein concentrations were measured using a Pierce™ BCA Protein Assay Kit (Thermo Fisher Scientific, Israel) according to the manufacturer’s protocol. The expression levels were evaluated using 10% Sodium Dodecyl Sulfate-Polycrylamide Gel Electrophoresis (SDS-PAGE).

### Enzymatic activity assay

Hexokinase catalytic activity was measured using an enzymatic coupled assay using a previously described method (Feng et al., 2015). The conversion of D-glucose to D-glucose-6-phosphate is linked to the reaction of glucose 6-phosphate dehydrogenase that consumes NADP^+^ to form NADPH. All assays were performed in a total volume of 100 µL, containing 100 mM Tris pH 7.5, 10 mM MgCl_2_, 1 mM NADP^+^, 1 mM ATP, and ~5 µg of NADP^+^-dependent glucose-6-phosphate dehydrogenase from *E. coli*. This enzyme was expressed in our lab following the protocol described elsewhere using the vector kindly provided by Prof. Ron Milo (Weizmann Institute of Science, Rehovot, Israel). The reaction was initiated by the injection of a range of D-glucose concentrations (0, 0.001, 0.01, 0.05, 0.1, 0.2, 0.5, 0.8, and 1 mM) in duplicate in a transparent 96-well plate (Greiner) to the reaction mixture with a constant concentration of purified hexokinase enzyme (10 µg). Reactions were carried out at +30°C, and absorbance at 340 nm was recorded continuously to monitor the production of NADP^+^ using a plate reader (Infinite^®^ 200 PRO; TECAN). For kinetic analysis, the steady-state rate (Abs_340_/s) was determined for each reaction by plotting a straight line over the first 30 s. The average rate with standard deviation was plotted for each D-glucose concentration and fitted to a Michaelis-Menten equation to calculate the kinetic parameters using an NADP^+^ extinction coefficient of 6.220 M^-1^ cm^-1^. The calculations were executed, and visual representations were produced utilizing GraphPad Prism version 9.5.1.

### AlphaFold 3D structure predictions and docking simulations

Ghxk9 structure prediction was carried out using AlfaFold2 (https://colab.research.google.com/github/sokrypton/ColabFold/blob/main/AlphaFold2.ipynb#scrollTo=kOblAo-xetgx) (Jumper et al., 2021) using the standard parameters. The structure was validated by direct comparison with the X-ray diffraction-resolved structure (resolution 2.001 Å) of hexokinase 1 from *Arabidopsis thaliana* (AtHxk1, pdb: 4QS7) (Feng et al., 2015). The pdbs from the 3D structures of GhHkx9 and AtHxk1 structures were overlaid, analyzed, and visualized using UCSF Chimera v1.15 (Pettersen et al., 2004). The root mean square deviation (RMSD) was calculated from the SuperPose Version 1.0 webserver (http://superpose.wishartlab.com/). D-glucose or 2-NBDG was docked into the GhHkx9 active site Autodock Vina (Trott and Olson, 2010). Autodock Tools (Morris et al., 2009) was used to add charges and autodock types and convert GhHkx9, D-glucose, and 2-NBDG into pdbqt formats. A grid box size of 70 × 70 × 70 Å centered at X = -0.109, Y = 9.551, and Z = 94.662, was defined to ensure unrestricted ligand movement and cover the entire active site. Other parameters were set as follows; number of maximum binding modes = 50, exhaustiveness value = 64, and the maximum energy difference between the worst and best binding mode = 4 Kcal/mol, respectively. The RMSD value for the re-docked conformation was calculated employing the online DockRMSD server (Bell and Zhang, 2019). All structures were visualized using PyMol version 2.5.2 (The PyMOL Molecular Graphics System, Version 2.0 Schrödinger, LLC) (Delano, 2002) and/or UCSF Chimera v1.15 (Pettersen et al., 2004).

## Results

### Phenotypic impact of exogenously feeding 2-NBDG on fertilized cotton ovules *in vitro cultures*

**Figure 1:**
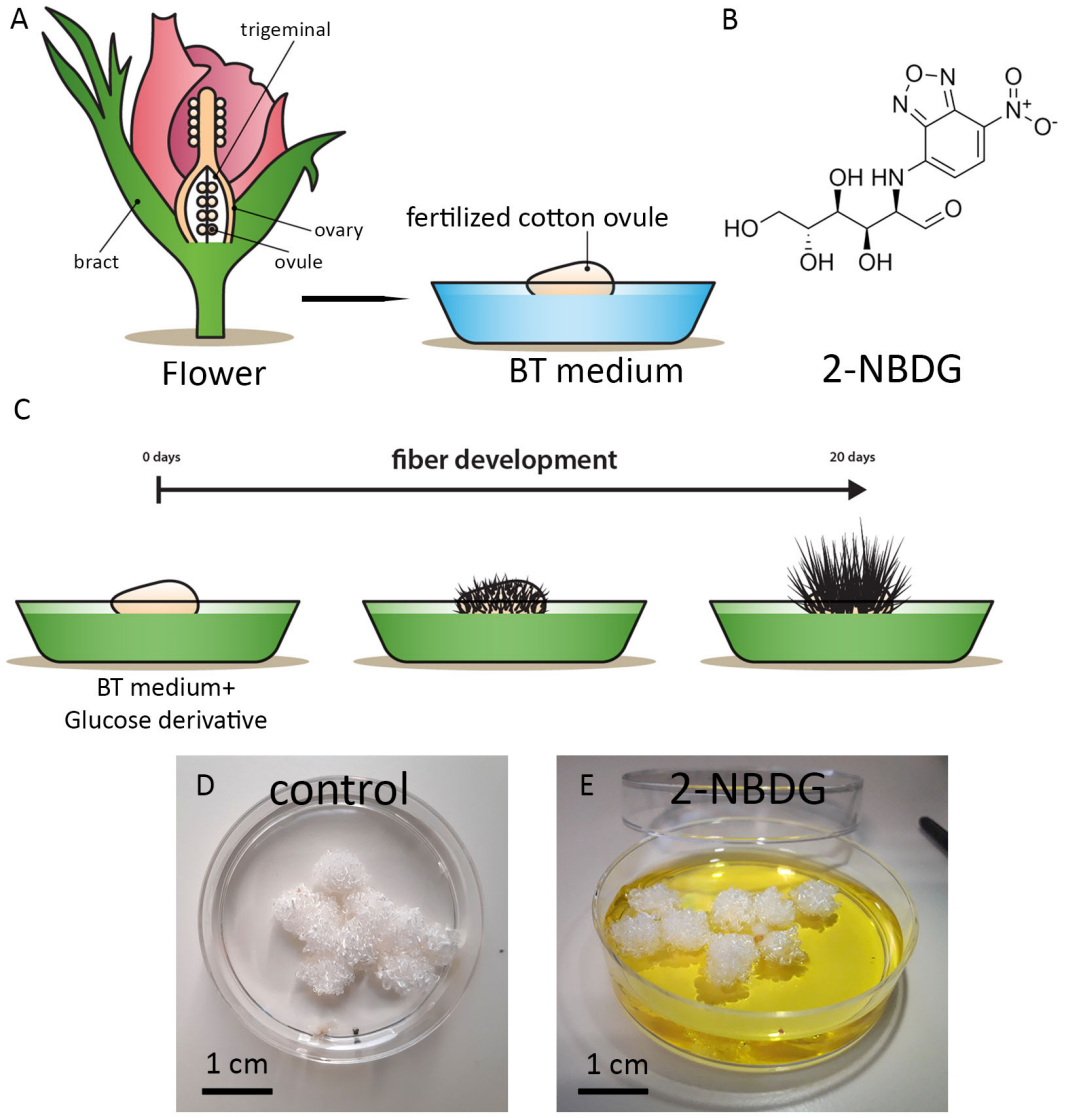
**(A)** Schematic representation of the cotton flower and fertilized *in vitro* cotton ovule cultures. **(B)** Chemical structure of 2-(N-(7-nitrobenz-2-oxa-1,3-diazol-4-yl)amino)-2-deoxyglucose) (2-NBDG). **(C)** Schematic representation of fertilized cotton ovule *in vitro* cultures supplemented with glucose derivatives. **(D, E)** Representative photographs of fertilized cotton ovules *in vitro* cultures after 20 days of growth under standard experimental conditions. Control **(D)**, treated with 2-NBDG **(E)**. No apparent macroscopic morphological alterations are observed.

To explore the effect of 2-NBDG on cotton ovule and fiber growth and development, 2-NBDG was added exogenously to the growth medium containing floating fertilized cotton ovules (**Figures 1A–C**). After this standard incubation period, no apparent macroscopic morphological alterations in ovules and fibers development were observed between the fertilized cotton ovule *in vitro* cultures grown in the absence (control ovules, henceforth) (**Figure 1D**) or in the presence of 2-NBDG (**Figure 1E**) (2-NBDG ovules henceforth). In addition, the fibers were not observed to display any yellow coloration as previously reported for fertilized *in vitro* cotton ovule cultures fed exogenously with 5(6)-carboxyfluorescein-glucose (Natalio et al., 2017).

### Spatial distribution of 2-NBDG in cotton ovules by confocal laser scanning microscopy analysis of its cross-sections

**Figure 2:**
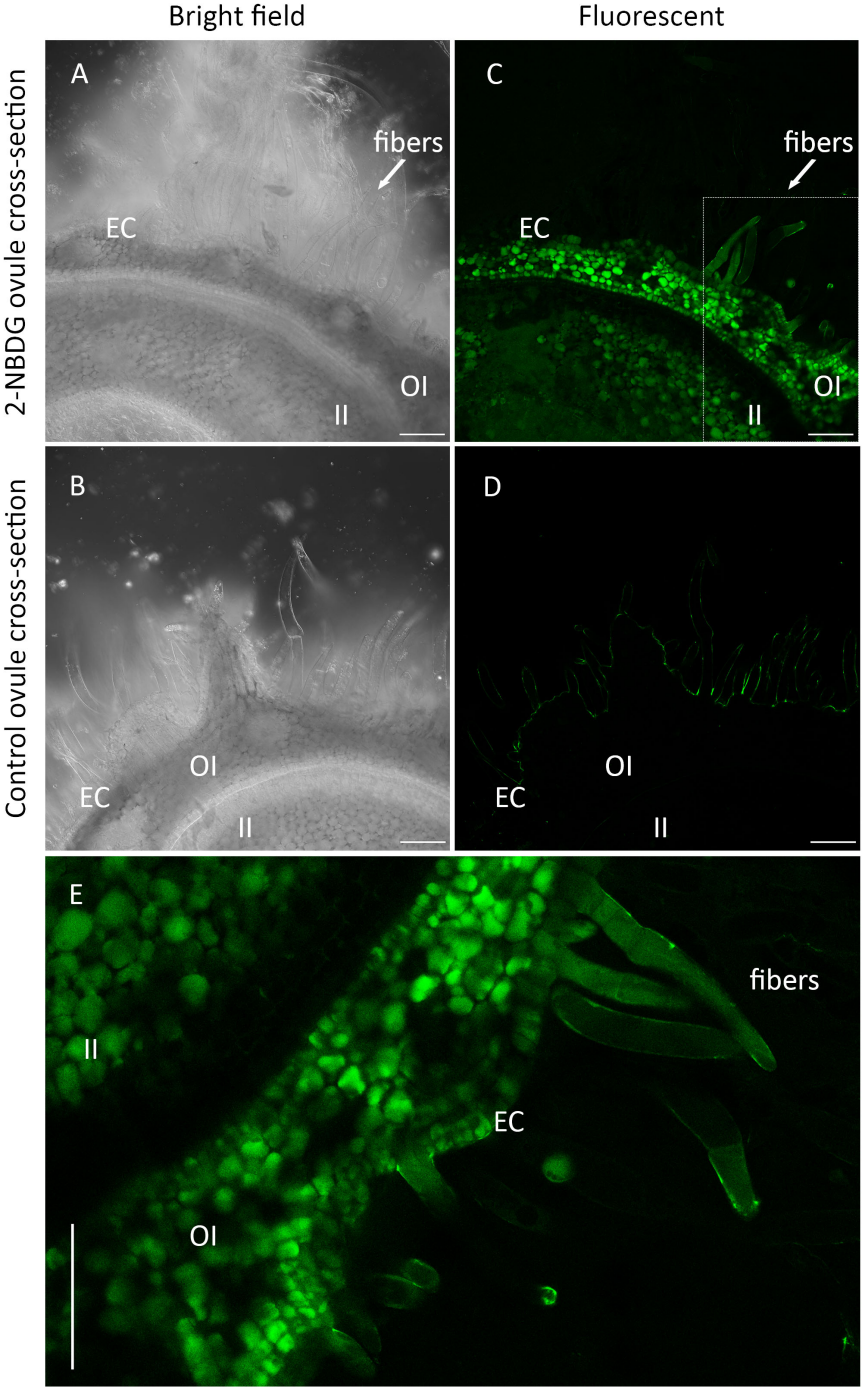
**(A, C)**, Representative Confocal Laser Scanning Microscope (CLSM) bright-field **(A)** and correspondent fluorescence **(C)** images of the outer-rim cross-sections of fertilized ovule *in vitro* cultures grown in the presence of 2-(N-(7-nitrobenz-2-oxa-1,3-diazol-4-yl)amino)-2-deoxyglucose) (2-NBDG), showing the inner integument (II), outer integument (OI), epidermal cells (ECs), and protruded ECs (fibers). A green fluorescent signal was found in multiple OI cells, ECs **(C)**, and fibers but not in their cell wall. **(B, C)**, Representative CLSM bright-field **(B)** and correspondent fluorescence **(C)** images of the outer rim cross-sections of fertilized ovules *in vitro* cultures grown in the absence of 2-NBDG, showing the same region of the tissues. No fluorescence was found in any of the tissues **(D, E)**, Zoom in image of **(C)** showing a green fluorescent signal was found inside some OI cells, ECs, and fibers but not in the cell wall. Some ECs are multicelled as a result of continued cell division. Scale bars: 100 µm.

Confocal laser scanning microscopy (CLSM) bright field images of cross-sections of the control ovules and 2-NBDG ovules show the inner integument (II), outer integument (OI), epidermal cells (ECs), and the protruded ECs (henceforth, fibers) (**Figures 2A, B**). No morphological changes are apparent between cross-sections of control ovules and 2-NBDG ovules, as reported for the cotton ovules incubated with 2-deoxy-2-iodo-D-glucose (Natalio, 2020). In the 2-NBDG ovules, there is an evident fluorescent signal accumulation in multiple cells in the outer integument (OI) with different accumulation levels (**Figure 2C**, OI). In contrast, a fluorescent signal is mostly uniformly distributed in the inner integument (II) (**Figure 2C**, II), with a lower fluorescence signal in the tissues between II and OI. The presence of a fluorescent signal was also found in some ECs and fibers (**Figure 2C**, fibers), but no fluorescence was found at the fibers’ cell wall. Some ECs are multicelled as a result of continued cell division (Hof and Saha, 1997; Saha and Hof, 2005). No green fluorescence signal was found in the cross-sections of control ovules imaged under the same settings (**Figure 2D**). **Figure 2E** displays a detailed fluorescent microscopic image from a region selected from the cross-section presented in **Figure 2C**, where a fluorescent signal is clearly observed inside some OI cells, ECs, and fibers but not at the fiber’s cell wall.

### Metabolic profile of cotton fibers excised from ovules fed with 2-NBDG reveals glycolysis inhibition and accumulation of D-ribose

**Figure 3:**
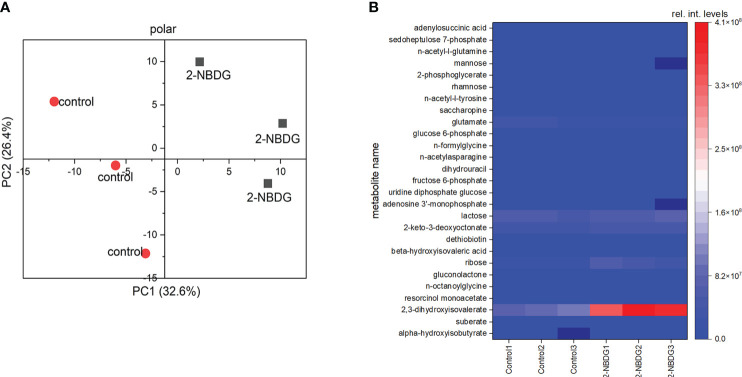
**(A)** Principal Component Analysis (PCA) plot based on all putatively identified polar compounds identified from 2-NBDG fibers extracts (grey squares) *vs*. control fibers extracts (red circles). **(B)**, Heatmap showing the accumulation or reduction of the 27 statistically significant (*p-value*<0.05) polar metabolites in their relative intensity levels between 2-NBDG fibers and control fibers.

Metabolic profiling of control and 2-NBDG fibers resulted in the putative identification of 225 metabolites (**Table S1**). 2-NBDG was detected in the fiber extracts, indicating that it reaches the cytosol, in agreement with CLSM analysis (**Figures 2C, E**). The score plot from a Principal Component Analysis (PCA) model calculated from all identified metabolites in the dataset (**Table S1**) shows a clear separation among polar metabolites between control and 2-NBDG fibers (**Figure 3A**). Twenty-seven metabolites displayed significant differences (*p-value*<0.05) in their relative levels between control and 2-NBDG fibers. Out of these twenty-seven statistically different metabolites, eleven were elevated, and sixteen were reduced (at least 1.75 times) in 2-NBDG compared to control fibers (**Table S2**). Heatmaps of relative intensity levels for these 27 metabolites in 2-NBDG *vs*. control fibers show a more pronounced increase in the relative intensity levels of 2,3-dihydroxyisovalerate, 2-keto-3-deoxyoctonate, and ribose and a decrease of L-glutamate (**Figure 3B**). Among these, alpha-hydroxyisobutyrate shows the highest accumulation, whereas adenylosuccinic acid shows the strongest reduction.

**Figure 4:**
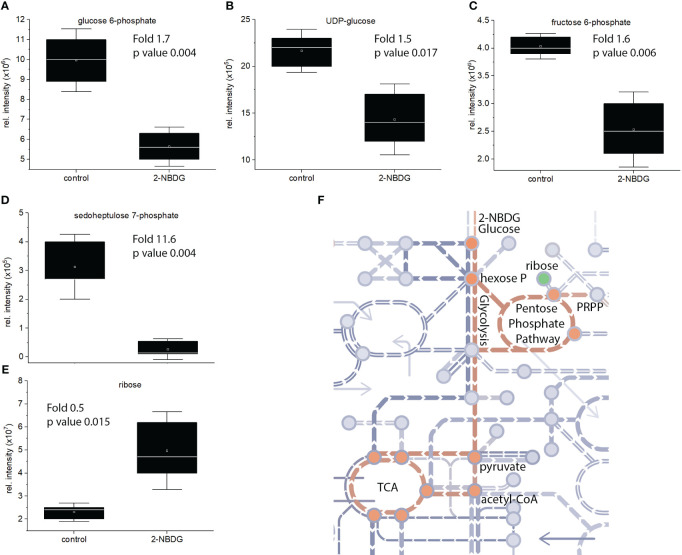
**(A-E)**. Individual box-and-whisker plots from the intensity levels of five statistically significant metabolites - glucose-6-phosphate **(A)**, UDP-Glc **(B)**, fructose-6-phosphate **(C)**, sedoheptluose 7-phosphate **(D)** and ribose **(E)** - between 2-NBDG *vs*. control fibers. Whiskers represent the standard deviation (SD). **(F)**, schematic representation of the metabolic pathway maps in metro-style from *Gossypium hirsutum* fibers affected by the presence of 2-NBDG. The green circle represents a metabolite (ribose) related to the glycolysis and glycolysis-related pathways. This metabolite was significantly upregulated compared to control fibers. Orange circles represent metabolites and respective pathways (glycolysis and TCA cycle) significantly downregulated compared to control fibers.

Focusing on the identified metabolites belonging to the glycolysis pathway, a reduction of D-glucose 6-phosphate (**Figure 4A**), UDP-glucose (**Figure 4B**), and D-fructose 6-phosphate (**Figure 4C**) by 1.7, 1.5. and 1.6 fold was found, respectively. Sedoheptluose 7-phosphate is reduced by 11.7-fold (**Figure 4D**), but ribose is increased by 0.5-fold (**Figure 4E**). These results suggest the downregulation of glycolysis and upregulation of energy-related pathways as compensatory mechanisms, as represented in **Figure 4F**.

### Heterologous expression of recombinant *G. hirsutum* hexokinase, catalytic activity assessment against glucose and 2-NBDG and docking simulations

**Figure 5:**
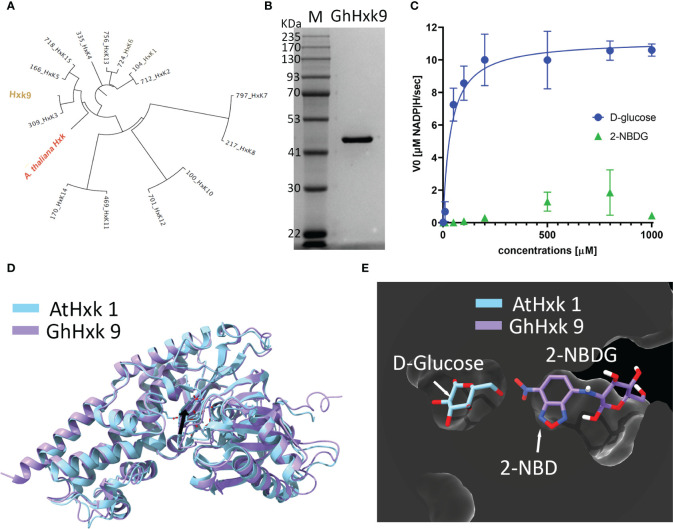
**(A)**, Phylogenetic tree was built from 15 identified hexokinases identified after *G. hirsutum* wide genome screening. The tree includes the *A. thaliana* hexokinase 1 (AtHxk1) sequence as an outgroup, previously described as heterologously expressed in the active form (Feng et al., 2015). **(B)**, Image of the Coomassie Blue-stained SDS-PAGE (10%) stained with showing successful heterologous expression and purification of GhHxk9 with a molecular weight of 51 kDa. **(C)**, Reaction rates obtained for recombinant GhHxk9 in the presence of increasing concentrations of D-glucose (blue circles) and 2-NBDG (green triangles) (0, 0.001, 0.01, 0.05, 0.1, 0.2, 0.5, 0.8, and 1 mM). The reaction rates were obtained by measuring the formation of NADP^+^ spectrophotometrically at 340 nm. Data obtained for D-glucose fitted to a Michaelis-Menten curve, from which the kinetic parameters K_m_ and V_max_ were calculated. In contrast, 2-NBDG (Green triangles) shows a linear relation and reduced catalytic activity compared to the natural substrate (blue circles) **(D)**, 3D superimposition X-ray diffraction-resolved structure of AtHxk 1 in glucose-bound form (light blue structure, pdb: 4QS7 (Feng et al., 2015) and AlphaFold predicted relaxed 3D structure of GhHxk9 (purple structure) with an RMSD of 1.10 Å. Black arrow points to the active site. **(E)**, 3D superimposition of AtHxk1 in glucose-bound form displaying the D-glucose (D-Glc) ligand in the active site and 2-NBDG ligand positioned in the GhHxk9 active site after docking simulation studies showing that 2-NBDG might have a distinct active site, thus, supporting the differences observed in the kinetic behavior.

*G. hirsutum* genomic screening yielded 15 entries for hexokinases (Hxk) (see supporting information for all the hexokinase gene and amino acid sequences) (Dou et al., 2022). Among the different hexokinases selected based on our criteria of phylogenetic proximity (**Figure 5A**) with *Arabidopsis thaliana* hexokinase 1 (AtHxk1) (Feng et al., 2015), GhHxk9 showed the highest expression yields. Sodium Dodecyl Sulphate-Polyacrylamide Gel Electrophoresis of the purified GhHxk9 shows a single band with a molecular weight of 51 kDa, matching the theoretical molecular weight value (GhHxk9 = 51 kDa) (**Figure 5B**). Th Kinetic analysis of the recombinant GhHxk9 displayed typical Michaelis-Menten behavior (**Figure 5C**, blue circles) under different concentrations of D-glucose from which an apparent K_m_ of 37 ± 19.8 µM, V_max_ = 11.23 ± 1.03 µM, K_cat_ of 8.9 s^-1^and K_cat_/K_m_ of 241 mM^-1^s^-1^ were calculated. No inhibition of the catalytic rates was observed for the concentration ranges used. The K_m_ for D-glucose calculated for GhHxk9 is approximately half the K_m_ calculated for the AtHxk1 for D-glucose (79 ± 12 µM) (Feng et al., 2015).

Replacing the D-glucose with 2-NBDG as a substrate for recombinant GhHxk9 resulted in an approximately five-fold decrease in reaction rates (**Figure 5C**, green triangles) compared to the rates obtained for D-glucose (**Figure 5C**, blue circles). The recombinant GhHxk9 shows linear behavior with 2-NBDG, contrasting the Michaelis-Menten behavior observed for the natural substrate. At a concentration of 1 mM of 2-NBDG, the catalytic activity is further reduced compared to other 2-NBDG concentrations and D-glucose.

To obtain a theoretical insight into the transition from a Michaelis-Menten to a linear behavior of recombinant GhHxk9 in the presence of D-glucose or 2-NBDG, respectively, docking simulations were performed using AlphaFold predicted relaxed 3D structure of GhHxk9 (henceforth 3D structure of GhHxk9) (**Figure S2**) and 2-NBDG. The AlphaFold predicted relaxed 3D structure of GhHxk9 shows high prediction confidence in general, except for the four regions: the C- and N- terminus and between L398 to K404 (TLKDGEK) and between S132 to R141 (SEGLHFSPDR) (**Figure S1**). The 3D structure of GhHxk9 predicted by AlphaFold overlapped with the X-ray diffraction-resolved structure of AtHxk 1 in glucose-bound form (Feng et al., 2015), resulting in a minimal root mean square deviation (RMSD) of 1.10 Å between 455 amino acids Cα (**Figure 5D**). After analyzing the AtHxk1 structure and amino acid sequence alignment, the active sites amino residues for GhHx9 have been identified as T167, K168, N202, D203, N229, E257, and E288. These correspond to the residues T194, K195, N229, D230, N256, E284, and E315 previously identified in AtHxk 1 (Feng et al., 2015). The identified active site amino acids from GhHxk9 and AtHxk1 spatially overlap with an RMSD of 0.374 Å between 7 amino acids Cα (**Figure S2**). Molecular docking simulations performed between GhHxk9 and 2-NBDG show that 2-NBDG is not located at the active site as the D-glucose in the AtHxk1 crystal structures. Also, the fluorescent tag from the 2-NBDG (2-NBD moiety) is found inside the active site pocket, leaving the glucose moiety outside (**Figure 5E**).

## Discussion

In recent years, the utilization of exogenous fluorescent glucose derivatives has emerged as a potent tool for investigating various cellular processes in plants, such as, for example, elucidating sugar transport mechanisms using esculin (Knoblauch et al., 2015; Rottmann et al., 2018; Rottmann and Stadler, 2019). However, comparatively less attention has been directed toward different fluorescent glucose derivatives and exploring their influence on glucose-related metabolism (Etxeberria et al., 2005; Conde et al., 2007; Tian et al., 2023), including cell wall-related phenomena. It is important to note that glucose derivatives, while sharing similar structural properties to glucose, often possess altered chemical characteristics and exhibit different interactions with organisms, emphasizing that glucose derivatives should be considered analogs of glucose rather than identical compounds (Jang and Sheen, 1994; Kurtoglu et al., 2007; Fatangare et al., 2015; Fatangare and Svatoš, 2016; Van Scherpenzeel et al., 2022).

In this study, fertilized cotton ovule *in vitro* cultures (Beasley, 1973; Beasley and Ting, 1974; Haigler et al., 1991; Triplett, 2000; Natalio et al., 2017) were fed exogenously with 2-NBDG, follows by a spatial distribution assessment within the different tissues of cotton ovules. CLSM imaging of cross-sections of the outer rim of cotton ovules grown in the presence of 2-NBDG showed a non-uniform accumulation of green fluorescence in the different tissues, i.e., in the OI fluorescent signal was observed at varying intensity levels in different cells while it was completely absent in other OI-composing cells. A similar heterogeneous distribution of fluorescent signal was also observed in epidermal cells (ECs) and fibers, although no fluorescent signal was found in the cell wall (**Figures 2C, E**).

In accordance with previous studies (Ruan et al., 2001; Etxeberria et al., 2005; Conde et al., 2007), the distribution of fluorescent signal within cells in the OI appears to be localized to the vacuole (**Figure 2E**). Differential 2-NBDG accumulation between cells in the OI could be attributed to a higher degree of cell-cell traffic regulation in this tissue, achieved through plasmodesmata gating(Ruan et al., 2001). The fluorescent signal was also observed in the II when the signal was stronger towards the center of the ovule and fading gradually towards the OI. Interestingly, in large areas of the II, the distinction between cells is unclear, suggesting a lack of cell-cell segregation and vacuole sequestration in this tissue.

Interestingly, it was reported that loading a phloem-mobile symplastic fluorescent probe, carboxyfluorescein-ester (CF) by the shoots cut ends led to a different distribution pattern, where CF was restricted to the outer integument, suggesting active transport of 2-NBDG into the inner integument (Ruan et al., 2001; Wang and Ruan, 2012). An alternative explanation for 2-NBDG signal distribution could be that uptake from the medium occurs in cells belonging to the OI that are in direct contact with the growth medium. This uptake process might occur actively by a plasmalemma-bound carrier-mediated system or be mediated by a glucose-repressible, H^+^-dependent active saturable transport system and/or an endocytic/diffusional component transport process as proposed for Sycamore (*Acer pseudoplatanus L*.) cells or *O. europaea L.* var. *Galega Vulgar* cell suspensions (Etxeberria et al., 2005; Conde et al., 2007). 2-NBGD is then transported apoplastically through cells belonging to the OI, in line with the findings for carboxyfluorescein accumulation in the ovules after loading through the shoot cut end (Ruan et al., 2001). However, this seems unlikely as there is no fiber development in the ovules’ cells immersed region, and consequently, all fibers should exhibit staining at the cell wall.

The accumulation of 2-NBDG in the fibers’ cytosol, in contrast to its absence from the cell wall, might suggest that glycolytic enzymes, such as hexokinases, metabolize 2-NBDG at a reduced rate compared to D-glucose or not metabolize it altogether.

Chromatographic analysis of 2-NBDG fibers’ extracts showed the presence of 2-NBDG in the cytosol, confirming the CLSM imaging performed on the cross-sections of cotton ovules’ outer rim (**Figures 2C, E**). However, the detection of 2-NBDG derivatives, such as, for example, 2-NBDG 6-phosphate, 2-NBDG 1-phosphate, and UDP-2-NBDG, is challenging due to the absence of standards and the numerous compounds typically present in whole extract chromatograms (e.g., m/z search). Untargeted metabolic profiling and data analysis with a primary focus on metabolites belonging to the glycolysis pathway revealed a reduction of some of these metabolites. Specifically, a reduction of D-glucose 6-phosphate (**Figure 4A**), UDP-glucose (**Figure 4B**), and D-fructose 6-phosphate (**Figure 4C**) was found. Despite the reduction in UDP-glucose levels, there is no apparent alteration/inhibition of fibers and ovule development in the 2-NBDG fibers (**Figure 1E**). Fructose 6-phosphate is a direct product of the catalytic conversion of D-glucose 6-phosphate by glucose-6-phosphate isomerase and a key metabolite in the Pentose Phosphate Pathway (PPP). It is converted to sedoheptluose 7-phosphate by a transaldolase. Thus, the reduction of D-glucose 6-phosphate will lead directly to a decrease in fructose 6-phosphate levels and, consequently, to reduced levels of the sedoheptluose 7-phosphate (**Figure 4D**), resulting in the replenishment of carbons into glycolytic pathways. The relative abundance of D-ribose increases (**Figure 4E**) in 2-NBDG fibers compared to control fibers. Currently, there is no well-established major pathway that makes use of unphosphorylated ribose (Riggs et al., 2016). D-ribose 5-phosphate originates from multiple metabolic routes within the PPP and is one of the potential precursors of ribose. The observed accumulation of D-ribose could suggest the dephosphorylation of D-ribose 5-phosphate and compensate for an energetic unbalance created by the downregulation of glycolysis, as summarily depicted in **Figure 4F**.

Based on untargeted metabolic profiling of the 2-NBDG and the identification of 2-NBDG inside some fibers revealed by microscopic and chromatographic analyses, we hypothesized that the 2-NBDG interacts with *G. hirsutum* hexokinases resulting in metabolic downregulation regulatory bottleneck. To investigate this hypothesis, theoretical and experimental approaches were adopted by combining catalytic activity assessment of heterologously expressed recombinant active hexokinase from *G. hirsutum* against 2-NBDG and docking modeling studies. The findings from this integrated approach provided preliminary support to our initial hypothesis, as the presence of 2-NBDG resulted in a reduction in the catalytic rates of recombinant GhHxk9, suggesting potential interaction with a distinct active site. However, it remains uncertain whether 2-NBDG exerts an allosteric effect on GhHxk9.

## Conclusions

A fluorescent glucose derivative (2-NBDG), commonly employed to monitor glucose-related biological processes *in vivo*, was added exogenously to cotton ovule *in vitro* culture models, thereby allowing visualization of its spatial distribution within the ovules’ cross-sections with a tissue-specific accumulation and differing concentrations within cells belonging to the same tissue. Certain fibers exhibited a fluorescent signal in the cytosol but not in the cell wall. Metabolic profiling of the fibers and complementary biochemical *in vitro* validation offer indirect evidence supporting the hypothesis that the interaction between 2-NBDG and hexokinase downregulates central carbon metabolism, including glycolysis pathway and related pathways with upregulation of alternative pathways as counter-response. However, direct evidence detailing how 2-NBDG might be metabolized or catabolized and its interaction with enzymes from other pathways, including glycolysis, leading to their up or downregulation, remain to be elucidated in future research.

Finally, fluorescent glucose derivatives are ready-to-use analogs for tracking glucose-related biological processes that interact differently than the natural substrate for each tissue within an organism. Direct comparison of their mode(s) of action and extrapolation into biochemical consideration should go beyond microscopic inspection and include complementary analytical techniques.

## Data availability statement

The original contributions presented in the study are included in the article/**Supplementary Materials**. Further inquiries can be directed to the corresponding author. The data presented in the study are deposited in the public github repository https://github.com/fnatalio/2-NBDG-.

## Author contributions

MS performed genome mining, enzyme expression, and purification, kinetic data analysis. Contributed to original and final manuscript preparation. SS performed the enzyme expression and purification, and kinetic measurements, and contributed to the original and final manuscript preparation with text and images. O-AK performed the culture of cotton in the greenhouse, in-vitro cotton cultures with 2-NBDG, cross-sectioning, CLSM imaging and data processing, and contributed to the original and final manuscript preparation with text and images. MI performed the extraction of polar metabolites from the cotton fibers metabolomic profiling, and data analysis, and contributed to the original and final manuscript preparation with text and images. SM performed the supervision of metabolomics data analysis, and contributed to the original and final manuscript preparation with text and images. FN wrote the draft and final manuscript. He was responsible for the supervision and coordination of the project and acquisition of the funding. All authors contributed to the article and approved the submitted version.
