# Supplementary material for: 2-NBDG Uptake in Gossypium hirsutum in vitro ovules: exploring tissue-specific accumulation and its impact on hexokinase-mediated glycolysis regulation
Source: Front Plant Sci. 2023 Sep 25;14:1242150. doi: 10.3389/fpls.2023.1242150 (PMC10561253; doi:10.3389/fpls.2023.1242150)
Supplement: Supplementary file 1 [file DataSheet_1.pdf]

Supplementary Information

2-NBDG Uptake in *Gossypium hirsutum* In Vitro Ovules: Exploring Tissue-Specific Accumulation and Its Impact on Hexokinase-Mediated Glycolysis Regulation

Melina Shamshoum[1,#], Ofir-Aharon Kuperman[1,#], Sapir Korman Shadmi[1], Maxim Itkin[2], Sergey Malitsky[2], and Filipe Natalio[1]*

[1] Department of Plant and Environmental Sciences, Weizmann Institute of Science, Rehovot 7610001, Israel

[2] Metabolic Profiling Unit, Life Sciences Core Facilities, Weizmann Institute of Science, Rehovot 7610001, Israel

* Corresponding author: filipe.natalio@weizmann.ac.il

#Shared contributions

Keywords: glucose derivatives, cotton, ovules, hexokinases, 2-NBDG, central carbon metabolism

Table S1. List of relative intensities of putatively identified polar metabolites in cotton ECs excised from the cotton ovules of in vitro cultures fed with 2-NBDG *vs*. control. N/A – value below quantification level.

**Metabolite name**	2-NBDG1	2-NBDG2	2-NBDG3	Control1	Control2	Control3	Probability*
1,1,1-kestopentaose	4.80E+04	7.40E+04	2.10E+05	1.30E+04	3.50E+04	1.30E+05	D
10-hydroxydecanoate	2.50E+04	8.30E+03	5.10E+03	2.50E+04	5.20E+04	8.20E+03	D
1-aminocyclopropanecarboxylate	1.10E+06	1.10E+06	1.30E+06	1.20E+06	1.40E+06	4.70E+05	A
1-deoxy-d-xylulose-5-phosphate	1.90E+05	1.80E+04	2.80E+05	3.40E+05	6.90E+04	N/A	D
2-(4-hydroxyphenyl)propionic acid	1.90E+06	2.00E+04	1.50E+06	3.90E+04	1.90E+06	8.10E+04	B
2,3-dihydroxybenzoate	3.00E+05	5.10E+05	5.00E+05	3.60E+05	5.20E+05	1.90E+05	B
2,3-dihydroxyisovalerate	3.30E+08	4.10E+08	3.70E+08	8.10E+07	8.60E+07	9.70E+07	A
2,4,6-trihydroxybenzoic acid	1.30E+05	1.40E+05	8.30E+04	3.00E+05	N/A	1.50E+05	D
2',4'-dihydroxyacetophenone	7.10E+05	4.70E+05	8.50E+05	6.20E+05	6.90E+05	5.40E+05	A
2-aminoadipic acid	1.30E+06	2.10E+06	1.80E+06	1.60E+06	1.80E+06	2.10E+06	D
2-aminoisobutyrate	2.40E+06	3.80E+06	4.20E+06	1.10E+06	4.00E+06	4.20E+06	A
2-aminophenol	4.10E+05	9.70E+04	7.00E+05	2.00E+05	4.10E+05	4.40E+05	C
2-deoxy-glucose	1.80E+07	1.80E+07	1.60E+07	1.40E+07	1.60E+07	2.00E+07	B
2-hydroxypyridine	1.70E+06	6.80E+05	5.70E+05	1.40E+06	9.10E+05	1.20E+06	A
2-isopropylmalic acid	1.10E+06	2.50E+06	2.10E+06	7.10E+05	1.30E+06	6.10E+05	A
2-keto-3-deoxyoctonate	5.20E+07	4.60E+07	5.10E+07	3.40E+07	3.80E+07	3.70E+07	A
2-ketobutyric acid/acetoacetate	1.70E+06	3.00E+06	3.60E+06	1.80E+06	3.90E+06	2.80E+06	A
2'-o-methylguanosine	1.80E+05	2.40E+05	2.30E+05	3.80E+04	2.30E+05	2.10E+05	B
2-oxoadipate	3.30E+06	4.50E+06	4.20E+06	3.30E+06	6.90E+06	4.20E+06	A
2-phosphoglycerate	1.00E+05	1.60E+05	1.30E+05	3.70E+05	3.10E+05	3.70E+05	A
2-propenoate	8.00E+05	9.40E+05	7.70E+05	8.70E+05	7.10E+05	9.30E+05	A
3-(2-hydroxyphenyl)propanoate	3.40E+04	5.50E+05	4.50E+04	9.40E+04	2.40E+05	2.10E+05	D
3-(4-hydroxy-3-methoxy-phenyl)-propionic acid	2.30E+05	2.10E+05	1.90E+05	2.70E+05	1.90E+05	1.90E+05	B
3-amino-4-hydroxybenzoate	1.70E+06	2.80E+06	2.00E+06	1.40E+06	1.80E+06	3.10E+06	B
3-dehydroshikimate	8.70E+05	2.40E+05	6.40E+05	9.50E+05	4.80E+05	1.60E+05	A
3-hydroxybenzaldehyde	6.40E+06	6.40E+06	8.10E+06	9.60E+06	1.00E+07	7.30E+06	A
3-hydroxybenzoate	8.20E+04	7.70E+04	8.60E+04	1.00E+05	2.40E+05	1.20E+05	A
3-hydroxybenzyl alcohol	1.60E+05	6.40E+04	3.50E+05	2.20E+04	4.20E+05	1.30E+05	D
3-hydroxybutyric acid	7.00E+06	5.60E+06	6.90E+06	9.40E+06	6.70E+06	8.50E+06	B
3-hydroxymethylglutarate	1.20E+08	1.40E+08	8.60E+07	1.20E+08	1.80E+08	1.30E+08	A
3-methyl-2-oxobutyrate	6.70E+06	6.80E+06	5.60E+06	8.70E+06	8.20E+06	6.20E+06	B
3-methyl-2-oxopentanoic acid	1.30E+08	1.10E+08	9.20E+07	9.90E+07	1.80E+08	6.60E+07	A
3-methyl-l-histidine	6.20E+04	3.10E+04	5.20E+04	8.20E+04	3.20E+04	2.90E+04	D

4-acetamidobutanoate	6.50E+06	1.10E+06	1.80E+06	6.50E+06	2.30E+06	5.70E+06	A
4-hydroxy-3,5-dimethoxy-benzoic acid	5.00E+05	4.30E+05	8.20E+05	5.00E+05	6.20E+05	4.70E+05	B
4-hydroxy-3-methoxyphenylglycol	2.10E+05	3.00E+05	4.40E+05	3.70E+05	3.70E+05	1.80E+05	B
4-hydroxybenzoate	6.50E+05	8.00E+05	9.30E+03	1.10E+06	9.70E+05	7.90E+05	A
4-pyridoxate	2.30E+06	2.20E+06	2.30E+06	1.20E+06	3.30E+06	1.50E+06	A
5-methyluridine	4.40E+05	7.30E+05	8.50E+05	3.80E+05	6.60E+05	8.60E+05	A
6-hydroxynicotinate	6.20E+04	1.70E+05	4.70E+04	1.20E+05	2.10E+05	1.10E+05	A
ac-val-oh	3.00E+06	2.60E+06	3.90E+06	2.50E+06	3.70E+06	3.60E+06	A
adenine	1.60E+08	1.20E+08	1.50E+08	1.10E+08	1.50E+08	1.20E+08	A
adenosine	2.40E+07	3.60E+07	3.50E+07	1.30E+07	2.10E+07	3.10E+07	A
adenosine 2',3'-cyclic phosphate	7.50E+05	9.50E+05	2.00E+06	1.90E+06	1.40E+06	1.50E+06	A
adenosine 3'-monophosphate	1.50E+07	1.30E+07	1.30E+07	2.10E+07	2.10E+07	2.00E+07	A
adenylosuccinic acid	9.40E+03	8.20E+02	7.80E+02	1.10E+05	1.00E+05	4.90E+04	A
adonitol	8.40E+06	1.00E+07	1.10E+07	9.20E+06	8.80E+06	9.40E+06	A
alanine	5.80E+07	6.50E+07	6.40E+07	6.40E+07	6.30E+07	5.90E+07	A
allantoin	3.70E+06	3.50E+06	3.90E+06	4.20E+06	3.80E+06	4.10E+06	A
alpha-hydroxyisobutyrate	6.70E+05	8.00E+05	8.90E+05	1.70E+05	5.80E+04	N/A	A
anthranilate	3.10E+05	1.40E+05	3.30E+05	2.20E+05	6.00E+05	3.70E+05	B
arabinose	2.30E+07	3.10E+07	3.30E+07	2.60E+07	3.10E+07	3.70E+07	A
arginine	7.30E+07	9.70E+07	9.10E+07	8.10E+07	8.80E+07	1.00E+08	A
argininosuccinic acid	9.10E+05	1.10E+05	2.20E+05	1.70E+06	9.70E+05	6.30E+05	A
ascorbate	N/A	N/A	N/A	3.00E+07	2.10E+07	1.80E+08	A
asparagine	3.20E+08	3.30E+08	3.40E+08	3.70E+08	3.40E+08	3.20E+08	A
aspartate	2.00E+08	2.30E+08	2.60E+08	1.80E+08	2.00E+08	2.30E+08	A
azelate	2.50E+06	3.20E+06	2.70E+06	2.30E+06	2.40E+06	2.10E+06	A
benzenesulfonic acid	4.30E+03	3.60E+04	8.10E+03	9.40E+03	8.00E+03	1.30E+04	C
benzoate	2.20E+07	2.00E+07	2.10E+07	3.10E+07	3.00E+07	2.30E+07	A
beta-alanine	1.30E+07	1.40E+07	1.60E+07	1.20E+07	1.30E+07	1.40E+07	A
beta-glycerophosphate	2.80E+07	1.90E+07	2.80E+07	3.90E+07	3.30E+07	2.90E+07	A
beta-hydroxyisovaleric acid	3.70E+06	3.20E+06	3.20E+06	1.70E+06	2.50E+06	2.00E+06	B
betaine	8.00E+06	7.80E+06	8.50E+06	6.90E+06	3.30E+06	5.50E+06	A
beta-nicotinamide adenine dinucleotide	5.60E+05	5.90E+05	6.50E+05	6.60E+05	6.70E+05	1.00E+06	A
butanoate	7.00E+06	4.20E+06	5.60E+06	5.30E+06	9.30E+06	7.90E+06	A
caffeate	1.90E+05	2.90E+06	N/A	9.10E+04	6.90E+05	N/A	B
cis-4-hydroxycinnamic acid	1.50E+06	2.60E+06	1.50E+06	8.50E+05	1.50E+06	2.40E+06	A
cis-aconitic acid	1.00E+08	1.60E+08	1.40E+08	1.30E+08	1.30E+08	1.80E+08	A

citicoline	1.10E+06	1.20E+06	1.00E+06	1.50E+06	1.30E+06	1.30E+06	A
citramalate	2.10E+08	2.30E+08	2.40E+08	1.10E+08	2.00E+08	1.90E+08	A
citrulline	2.60E+05	3.00E+05	3.50E+05	3.10E+05	3.50E+05	2.70E+05	A
creatinine	7.50E+04	9.00E+03	6.20E+04	7.30E+04	1.70E+04	3.70E+04	B
cyclic gmp	6.90E+03	6.60E+05	1.00E+06	N/A	N/A	9.60E+05	C
cystine	1.10E+06	3.80E+05	9.10E+05	1.50E+06	9.70E+05	4.60E+05	B
cytidine	2.00E+07	2.40E+07	2.20E+07	1.70E+07	2.00E+07	2.40E+07	A
cytidine 2',3'-cyclic phosphate	7.70E+06	1.10E+07	1.00E+07	6.90E+06	7.70E+06	1.00E+07	A
d-chiro-inositol	3.60E+07	5.00E+07	4.30E+07	5.30E+07	6.50E+07	6.20E+07	A
dehydroascorbate	2.10E+06	2.60E+05	1.50E+06	7.70E+05	4.90E+05	3.80E+04	A
deoxyuridine	8.00E+04	6.80E+04	1.20E+05	2.70E+04	3.00E+05	2.50E+05	A
d-erythrose	2.90E+06	3.20E+06	4.30E+06	2.30E+06	2.50E+06	2.40E+06	D
dethiobiotin	5.00E+06	3.40E+06	3.90E+06	2.30E+06	4.30E+06	2.50E+06	B
dihydrouracil	2.70E+06	3.50E+06	2.50E+06	4.40E+06	4.60E+06	4.80E+06	B
dihydroxyacetone dimer	7.00E+04	9.50E+04	1.70E+05	1.20E+05	1.50E+05	1.40E+05	A
dihydroxyacetone phosphate	1.90E+04	N/A	3.20E+04	3.40E+05	4.70E+04	N/A	B
dimethylmalonic acid	3.00E+04	1.10E+06	1.20E+06	1.20E+06	1.60E+06	8.30E+05	A
d-ribose 5-phosphate	1.40E+05	5.10E+04	3.00E+05	3.30E+05	3.40E+05	3.80E+05	B
d-sedoheptulose	9.10E+06	5.30E+06	6.00E+06	1.10E+07	1.60E+07	1.00E+07	C
d-sorbitol	3.10E+07	3.30E+07	3.90E+07	3.40E+07	4.10E+07	2.60E+07	A
erythritol	3.30E+06	2.00E+06	1.90E+06	1.10E+06	3.20E+06	4.80E+06	A
erythrose 4-phosphate	N/A	5.50E+05	4.90E+05	N/A	N/A	5.70E+05	A
ethylmalonate	3.90E+07	5.40E+07	5.80E+07	4.20E+07	5.40E+07	6.40E+07	A
ferulic acid	6.90E+06	7.90E+06	4.60E+06	7.30E+06	6.30E+06	8.40E+06	B
fructose	9.50E+08	1.20E+09	1.30E+09	1.00E+09	1.10E+09	1.40E+09	A
fructose 6-phosphate	2.10E+06	3.00E+06	2.50E+06	4.20E+06	3.90E+06	4.00E+06	A
galactarate	2.20E+06	2.30E+06	3.30E+06	1.70E+06	2.20E+06	2.70E+06	A
gamma-aminobutyrate	4.90E+08	4.40E+08	4.30E+08	5.50E+08	5.20E+08	4.80E+08	A
gamma-glu-epsilon-lys	1.60E+06	1.40E+06	1.60E+06	1.80E+06	1.70E+06	1.60E+06	A
gamma-l-glutamyl-l-alanine	6.10E+06	5.70E+06	6.10E+06	5.60E+06	5.60E+06	5.40E+06	B
gluconate	6.10E+07	6.30E+07	7.80E+07	8.10E+07	8.00E+07	2.20E+08	A
gluconolactone	3.60E+05	3.60E+05	4.40E+05	2.20E+05	4.10E+05	4.20E+05	A
glucose	4.70E+08	6.20E+08	8.00E+08	5.20E+08	6.30E+08	6.20E+08	B
glucose 6-phosphate	6.30E+06	5.60E+06	5.00E+06	1.10E+07	1.00E+07	8.90E+06	A
glucuronate	1.80E+07	1.90E+07	2.20E+07	1.90E+07	1.60E+07	1.80E+07	A
glutamate	1.60E+07	2.00E+07	1.70E+07	3.80E+07	3.30E+07	2.60E+07	A
glutamine	1.30E+08	1.10E+08	1.60E+08	1.60E+08	1.40E+08	1.10E+08	A
glutathione oxidized	4.30E+05	7.00E+05	6.90E+05	3.00E+05	2.80E+05	6.00E+05	A

glycerate	1.50E+08	1.50E+08	1.40E+08	2.00E+08	1.70E+08	1.50E+08	A
glycerol	7.00E+07	1.00E+08	8.80E+07	6.00E+07	7.80E+07	9.20E+07	A
glycine	1.20E+07	1.40E+07	1.10E+07	1.60E+07	1.50E+07	1.60E+07	A
glycolate	1.10E+09	1.40E+09	1.40E+09	1.60E+09	1.20E+09	1.50E+09	A
glyoxylate	5.60E+06	7.30E+06	7.20E+06	7.10E+06	7.70E+06	8.20E+06	A
guanine	1.90E+07	1.10E+07	2.00E+07	1.90E+07	1.40E+07	1.20E+07	A
guanosine monophosphate	5.00E+05	5.40E+05	4.60E+05	5.60E+05	6.30E+05	8.20E+05	A
gulonic acid gamma-lactone	6.70E+05	1.00E+06	1.40E+06	6.10E+05	7.10E+05	3.70E+05	A
hexanoate	5.70E+06	2.80E+06	4.10E+06	3.60E+06	4.00E+06	2.20E+06	A
hydrocinnamic acid	4.00E+05	7.90E+04	4.20E+05	8.80E+04	1.60E+05	9.50E+03	B
hydroquinone	2.40E+05	3.20E+05	3.30E+05	3.40E+05	5.80E+05	3.30E+05	A
hydroxyphenyllactate	1.70E+05	7.40E+05	1.40E+05	2.50E+05	3.90E+05	2.20E+05	C
hydroxypyruvate	5.60E+07	5.20E+07	6.60E+07	5.60E+07	7.30E+07	6.00E+07	A
hypoxanthine	2.90E+06	3.70E+06	3.90E+06	4.30E+06	4.00E+06	4.20E+06	A
indole-3-carboxaldehyde	5.10E+05	2.20E+05	7.60E+05	2.20E+05	5.10E+05	1.10E+05	A
indole-3-carboxylic acid	6.90E+03	4.10E+03	3.50E+04	2.70E+04	1.40E+04	1.30E+04	A
indole-3-pyruvate	N/A	N/A	N/A	4.00E+04	1.00E+05	1.30E+05	C
isocitrate	2.30E+07	3.30E+07	2.40E+07	2.80E+07	3.20E+07	5.10E+07	D
isoleucine	4.70E+07	1.00E+08	9.20E+07	7.50E+07	5.60E+07	8.00E+07	C
isomaltotriose	2.00E+06	2.60E+06	2.60E+06	2.00E+06	1.70E+06	1.30E+06	A
isovaleric acid	1.30E+07	5.90E+06	6.00E+06	1.00E+07	2.10E+07	1.30E+07	A
itaconate	5.40E+06	9.00E+06	6.90E+06	6.70E+06	6.70E+06	8.10E+06	LA
lactic acid	1.50E+07	2.40E+07	2.10E+07	1.50E+07	2.00E+07	2.10E+07	A
lactobionic acid	3.50E+05	2.70E+05	5.90E+05	3.20E+05	4.30E+05	2.20E+04	A
lactose	6.80E+07	6.80E+07	8.00E+07	5.50E+07	5.70E+07	4.70E+07	A
leu-ala	4.40E+05	7.20E+05	4.40E+05	1.00E+05	4.30E+05	3.30E+05	B
leucine	3.00E+08	3.10E+08	2.80E+08	2.40E+08	3.00E+08	3.10E+08	B
leu-gly	3.40E+06	1.40E+07	1.10E+07	7.10E+06	4.00E+06	9.90E+06	A
lipoamide	8.30E+04	9.80E+03	2.50E+04	2.30E+05	4.90E+04	7.80E+03	B
l-methionine sulfoxide	9.20E+06	1.20E+07	1.50E+07	4.90E+06	1.30E+07	1.20E+07	A
lumichrome	3.80E+05	5.70E+05	7.20E+05	7.80E+05	4.10E+05	6.70E+05	A
lysine	3.20E+07	4.00E+07	3.70E+07	3.80E+07	3.70E+07	4.10E+07	D
maleamate	5.90E+05	7.00E+04	9.30E+04	3.80E+04	1.10E+05	5.30E+05	C
maltotriose	1.40E+07	1.60E+07	2.20E+07	1.70E+07	1.90E+07	2.40E+07	B
mannose	1.90E+05	2.10E+05	N/A	4.90E+05	3.20E+05	4.40E+05	A
meso-tartrate	6.20E+05	4.30E+05	3.40E+05	4.20E+05	5.60E+05	6.30E+05	A
mesoxalate	1.10E+06	4.60E+04	6.10E+05	6.70E+05	8.40E+05	7.50E+05	A
methionine	1.10E+08	8.90E+07	7.00E+07	1.10E+08	1.10E+08	1.30E+08	A

methyl vanillate	5.00E+04	1.90E+04	6.80E+04	5.10E+04	2.40E+04	2.00E+04	A
myo-inositol	3.60E+07	5.00E+07	4.30E+07	5.30E+07	6.50E+07	6.20E+07	B
n-(2-methylbutyryl)glycine	4.20E+06	1.50E+06	2.50E+06	1.60E+06	5.60E+06	2.30E+06	A
n-(phenylacetyl)glycine	8.70E+05	9.60E+05	1.30E+06	6.30E+05	1.40E+06	1.70E+06	D
n1-me-adenosine	2.10E+06	3.60E+07	1.10E+07	1.20E+06	3.30E+07	6.30E+07	C
n-acetylalanine	3.70E+06	5.10E+06	2.70E+06	2.00E+06	4.00E+06	2.50E+06	A
n-acetylasparagine	1.80E+06	1.30E+06	1.70E+06	2.30E+06	3.00E+06	2.70E+06	A
n-acetylglucosamine	1.00E+06	2.50E+06	8.20E+05	7.80E+05	1.90E+06	2.00E+06	B
n-acetylglutamate	1.80E+06	1.90E+06	2.10E+06	2.40E+06	2.60E+06	2.50E+06	B
n-acetylleucine	4.20E+06	1.90E+06	4.20E+06	1.20E+06	3.80E+06	1.00E+06	A
n-acetyl-l-glutamine	1.10E+05	1.40E+04	4.00E+04	3.50E+05	3.60E+05	6.00E+05	A
n-acetyl-l-methionine	1.70E+07	1.30E+07	9.00E+06	1.00E+07	1.20E+07	1.40E+07	B
n-acetyl-l-tyrosine	1.90E+05	1.30E+05	1.80E+05	2.90E+05	4.20E+05	4.30E+05	A
n-acetylphenylalanine	2.10E+06	9.90E+05	2.20E+06	8.50E+05	2.30E+06	9.30E+05	A
n-acetylserine	2.70E+06	2.00E+06	3.40E+04	3.50E+06	3.80E+06	4.30E+06	D
nepsilon-acetyl-l-lysine	8.10E+05	6.10E+05	7.30E+05	9.10E+05	7.20E+05	6.40E+05	A
nepsilon-trimethyllysine	5.90E+04	8.90E+04	1.00E+05	1.10E+05	1.10E+05	9.70E+04	B
n-formylglycine	7.50E+05	6.00E+05	5.00E+05	8.70E+05	1.10E+06	1.30E+06	C
n-formyl-l-methionine	2.60E+05	3.60E+05	4.10E+05	1.30E+05	6.10E+05	5.80E+05	A
nicotinamide	2.70E+06	3.10E+06	4.00E+06	2.40E+06	4.10E+06	4.30E+06	A
nicotinate	5.40E+06	4.20E+06	5.00E+06	3.30E+06	5.90E+06	5.50E+06	A
n-octanoylglycine	7.10E+05	5.70E+05	7.60E+05	3.70E+05	2.30E+05	1.90E+05	A
n-propionylglycine	1.70E+07	2.20E+07	2.30E+07	1.90E+07	2.10E+07	2.60E+07	C
o-hydroxyhippuric acid	4.60E+04	5.00E+04	5.60E+04	4.40E+04	1.50E+05	1.50E+05	LA
ophthalmate	1.20E+07	1.30E+07	1.50E+07	1.00E+07	1.10E+07	1.20E+07	B
o-succinyl-homoserine	1.20E+06	N/A	N/A	3.30E+05	8.80E+05	6.60E+05	A
oxalate	4.10E+07	3.70E+07	3.90E+07	2.80E+07	3.70E+07	3.90E+07	B
phe-gly	1.20E+06	1.90E+06	1.70E+06	8.70E+05	1.10E+06	1.40E+06	B
phenol	3.80E+06	2.80E+06	3.50E+06	3.50E+06	3.50E+06	3.30E+06	B
phenylacetate	1.20E+06	9.80E+05	1.30E+06	1.40E+06	8.70E+05	1.80E+06	B
phenylalanine	2.50E+08	3.00E+08	2.90E+08	2.80E+08	3.00E+08	3.30E+08	A
phenylpyruvic acid	2.60E+06	1.20E+06	2.90E+06	4.10E+06	3.60E+06	2.10E+06	A
phosphoenolpyruvate	1.10E+04	N/A	N/A	1.70E+05	3.60E+04	N/A	B
phosphoric acid	9.90E+07	1.60E+08	1.40E+08	9.20E+07	1.30E+08	1.80E+08	A
phosphorylcholine	3.20E+06	2.80E+06	3.40E+06	4.30E+06	3.80E+06	3.60E+06	A
pipecolate	9.60E+05	1.50E+06	1.80E+06	1.60E+06	1.70E+06	1.40E+06	A
pro-gly	2.70E+06	5.00E+06	3.90E+06	3.40E+06	5.60E+06	2.50E+06	A
proline	1.10E+08	1.20E+08	1.20E+08	1.30E+08	1.40E+08	1.30E+08	C

propionate	7.40E+06	7.00E+06	1.20E+07	6.00E+06	1.00E+07	5.30E+06	A
pseudouridine	2.70E+06	2.80E+06	2.90E+06	3.50E+06	2.90E+06	3.20E+06	A
pyridoxal	9.10E+04	1.50E+05	2.70E+05	1.10E+05	1.40E+05	1.90E+05	LA
pyridoxine	6.60E+06	8.30E+06	8.80E+06	7.70E+06	9.70E+06	1.00E+07	A
pyrrole-2-carboxylate	1.50E+06	1.40E+06	1.70E+06	1.20E+06	1.40E+06	1.50E+06	A
pyruvic acid	2.00E+08	1.50E+08	2.40E+08	2.80E+08	1.20E+08	N/A	A
quinate	6.00E+06	7.10E+06	5.20E+06	6.20E+06	5.80E+06	6.70E+06	A
resorcinol monoacetate	1.90E+05	2.30E+05	1.40E+05	3.90E+04	8.50E+04	2.90E+04	A
rhamnose	2.90E+06	2.40E+06	1.40E+05	4.80E+06	4.10E+06	5.60E+06	A
ribose	6.20E+07	4.70E+07	4.00E+07	2.00E+07	2.40E+07	2.50E+07	B
saccharopine	1.40E+05	1.50E+05	9.60E+04	2.50E+05	3.80E+05	2.30E+05	A
salicylamide	4.00E+05	4.70E+03	5.30E+05	6.00E+04	3.50E+05	2.50E+05	A
sedoheptulose 7-phosphate	5.40E+04	1.10E+04	1.40E+04	4.00E+05	2.70E+05	2.70E+05	D
serine	6.20E+07	6.40E+07	6.60E+07	8.10E+07	7.20E+07	7.00E+07	B
s-hexyl-glutathione	7.20E+04	6.00E+04	1.10E+05	7.50E+04	4.80E+04	8.20E+04	B
shikimate	3.50E+06	3.40E+06	3.90E+06	4.80E+06	4.40E+06	3.90E+06	A
sinapic acid	8.10E+05	9.80E+05	2.60E+05	9.50E+05	8.80E+05	1.20E+06	D
sorbate	4.00E+05	5.30E+05	7.00E+05	5.30E+05	2.90E+05	4.50E+05	C
stachyose	7.40E+05	5.10E+05	1.10E+06	5.50E+05	8.30E+05	7.80E+05	A
suberate	8.80E+05	8.90E+05	4.20E+05	5.40E+05	7.90E+04	5.10E+04	B
succinate	4.30E+08	4.10E+08	4.50E+08	6.40E+08	6.00E+08	5.40E+08	A
sucrose	2.60E+08	2.50E+08	2.70E+08	4.00E+08	4.30E+08	3.50E+08	A
taurine	7.70E+05	4.80E+05	1.30E+06	9.50E+05	4.10E+05	5.10E+05	C
thiamine	4.40E+06	3.00E+06	3.10E+06	5.40E+06	7.30E+06	2.40E+06	A
threonic acid	8.10E+07	5.90E+07	5.70E+07	1.20E+08	1.10E+08	5.10E+04	D
threonine	7.60E+07	8.20E+07	8.20E+07	9.10E+07	8.60E+07	8.30E+07	A
thymidine	1.40E+05	1.30E+05	2.50E+05	3.40E+04	2.90E+05	2.30E+05	A
trans-4-hydroxy-l-proline	2.30E+06	1.90E+06	2.00E+06	2.40E+06	2.50E+06	2.50E+06	A
trans-aconitate	1.20E+06	4.50E+06	1.60E+06	1.30E+06	1.80E+06	3.10E+06	A
tricarballylic acid	4.40E+05	2.90E+05	5.30E+05	2.80E+05	6.00E+05	5.60E+05	A
trigonelline	5.00E+05	7.80E+04	9.60E+04	1.70E+05	1.30E+06	4.70E+05	A
uracil	7.50E+07	5.30E+07	7.20E+07	8.20E+07	7.80E+07	5.60E+07	D
ureidopropionate	2.10E+06	1.30E+06	1.60E+06	1.90E+06	2.00E+06	1.90E+06	A
uric acid	N/A	N/A	N/A	3.40E+05	1.40E+05	2.30E+05	A
uridine	1.50E+08	1.90E+08	1.80E+08	1.20E+08	1.50E+08	1.80E+08	A
uridine diphosphate glucose	1.20E+06	1.70E+06	1.40E+06	2.20E+06	2.00E+06	2.30E+06	B
uridine diphosphate-n-acetylglucosamine	2.70E+06	3.00E+06	3.60E+06	3.20E+06	2.90E+06	3.20E+06	A

urocanate	3.90E+05	4.40E+05	2.70E+05	3.70E+05	3.60E+05	3.00E+05	A
valine	3.10E+08	3.70E+08	3.60E+08	3.30E+08	3.80E+08	3.90E+08	B
xanthine	6.80E+06	6.40E+06	5.40E+06	9.50E+06	7.20E+06	7.60E+06	B
xanthosine	N/A	1.60E+05	3.20E+04	1.40E+05	6.50E+04	1.60E+05	D
xanthosine-monophosphate	3.00E+04	8.10E+04	2.70E+04	4.30E+04	4.70E+04	1.20E+05	A

N/A – Not applicable (the levels are below the detection limit)

Probability

A Five criteria: accurate mass, RT, isotope match, fragment ions, and library match

B Four criteria: accurate mass, RT, isotope match, and fragment ions

C Four criteria: accurate mass, RT, fragment ions, and library match

D Three criteria: accurate mass, RT, and fragment ions

LA For low-molecular-weight metabolites without fragments in Library, Four criteria: accurate mass, RT, isotope match, and library match.

Table S2. List of the average intensity levels of twenty-seven polar metabolites found to be significantly ($p<0.05$) elevated or reduced (at least 1.75 times) in cotton ECs excised from the cotton ovules of in vitro cultures fed with 2-NBDG vs. controls.

Metabolite name	t-test *p*<0.05	relative level: control	relative level: NBDG	log2 ratio (treatment/control)
alpha-hydroxyisobutyrate	0.001	1.04E+06	1.11E+07	3.42
suberate	0.034	2.38E+00	1.10E+01	2.21
2,3-dihydroxyisovalerate	0.000	1.22E+09	5.19E+09	2.10
resorcinol monoacetate	0.011	7.03E+05	2.62E+06	1.90
n-octanoylglycine	0.006	3.64E+06	9.56E+06	1.39
gluconolactone	0.025	2.51E+07	5.53E+07	1.14
ribose	0.015	3.17E+08	6.95E+08	1.13
beta-hydroxyisovaleric acid	0.008	2.86E+07	4.73E+07	0.73
dethiobiotin	0.012	1.61E+08	2.24E+08	0.48
2-keto-3-deoxyoctonate	0.002	5.00E+08	6.97E+08	0.48
lactose	0.017	7.31E+08	1.01E+09	0.47
adenosine 3'-monophosphate	0.001	2.89E+08	1.92E+08	-0.59
uridine diphosphate glucose	0.017	3.04E+07	1.97E+07	-0.62
fructose 6-phosphate	0.006	5.53E+07	3.55E+07	-0.64
dihydrouracil	0.008	6.37E+07	4.06E+07	-0.65
n-acetylasparagine	0.010	3.69E+07	2.20E+07	-0.75
n-formylglycine	0.030	1.47E+07	8.66E+06	-0.76
glucose 6-phosphate	0.004	1.38E+08	7.93E+07	-0.80
glutamate	0.019	4.47E+08	2.45E+08	-0.87
saccharopine	0.024	3.97E+06	1.79E+06	-1.15
n-acetyl-l-tyrosine	0.010	5.21E+06	2.33E+06	-1.16
rhamnose	0.039	6.64E+07	2.55E+07	-1.38
2-phosphoglycerate	0.001	4.83E+06	1.81E+06	-1.41
mannose	0.030	5.76E+06	1.84E+06	-1.64
n-acetyl-l-glutamine	0.012	6.05E+06	7.74E+05	-2.97
sedoheptulose 7-phosphate	0.004	4.32E+06	3.71E+05	-3.54
adenylosuccinic acid	0.045	1.19E+06	5.16E+04	-4.53

N/A – Not applicable (the levels are below detection limit)
Probability
A Five criteria: accurate mass, RT, isotope match, fragment ions, and library match
B Four criteria: accurate mass, RT, isotope match, and fragment ions
C Four criteria: accurate mass, RT, fragment ions, and library match
D Three criteria: accurate mass, RT, and fragment ions
LA For low-molecular-weight metabolites without fragments in Library, Four criteria: accurate mass, RT, isotope match, and library match.

The amino acid and gene sequences of the 15 hexokinases found for *Gossypium hirsutum* after genome screening can be found in the *.xls file provided as supplementary information.

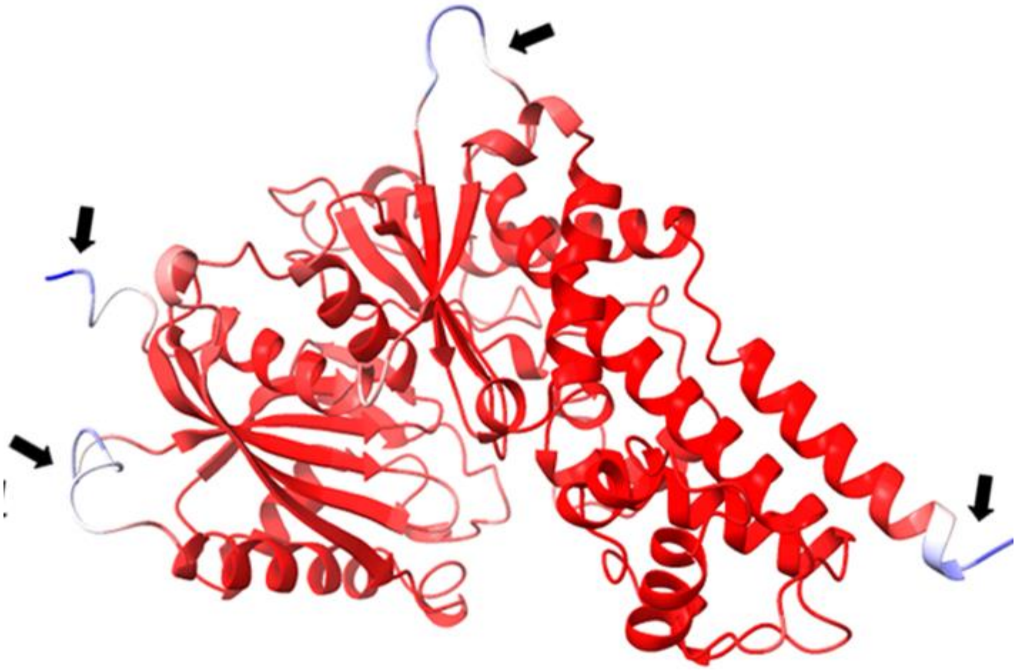

Figure S1. 3D representation of the Alpha-Fold-predicted structure for GhHxk9 showing an unfolded region at the C-and N-termini colored by B-factor. Black arrows point to low-prediction amino-acid regions in each structure, colored in blue and white. The low predicted region is composed of the following amino acid sequence: TLKDGEK (L398->K404). The low-predicted region on the left is composed of the following amino acid sequence SEGLHFSPDR (S132->R141). The GhHxk9 3D structure was analyzed, visualized, and rendered in Chimera X.[1]

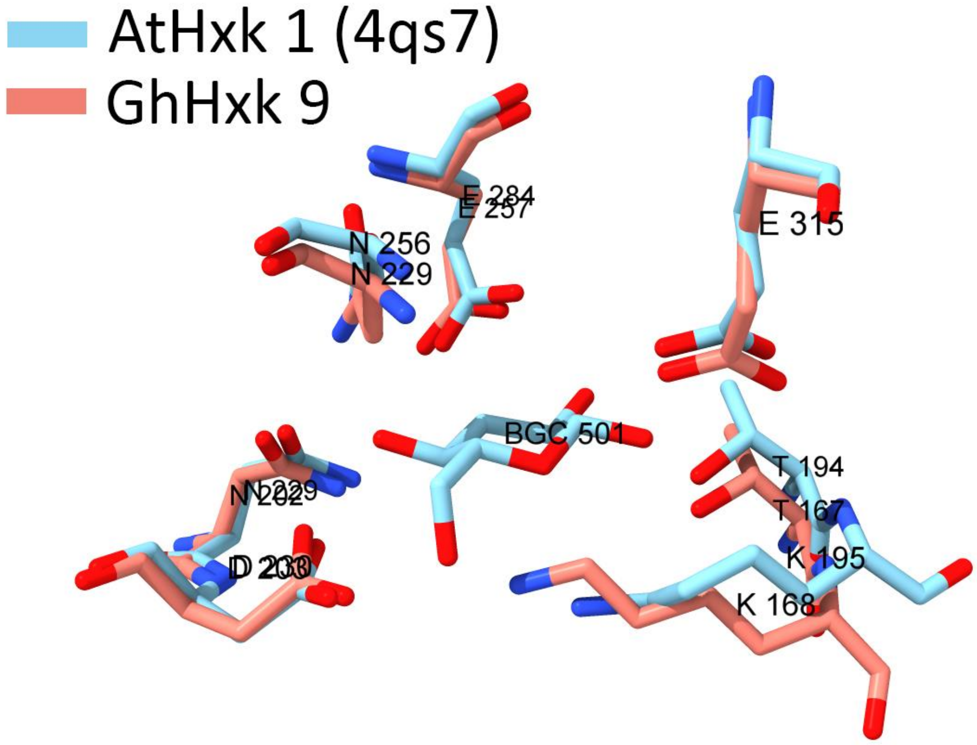

Figure S2. 3D superposition of selected active-site amino acids for AtHxk1 and GhHxk9, showing excellent spatial overlap with an RMSD of 0.374 Å between the Cα's of 7 amino acids. The structures were manipulated, analyzed, visualized, and rendered in Chimera X.[1]

References.

(1) Pettersen, E. F.; Goddard, T. D.; Huang, C. C.; Couch, G. S.; Greenblatt, D. M.; Meng, E. C.; Ferrin, T. E. UCSF Chimera—a visualization system for exploratory research and analysis. *Journal of computational chemistry* **2004**, *25* (13), 1605-1612.
